# Comparing marginal microleakage of three different dental materials in veneer restoration using a stereomicroscope: an *in vitro* study

**DOI:** 10.1038/bdjopen.2016.10

**Published:** 2017-01-06

**Authors:** Shuang Jia, Dong Chen, Defang Wang, Xiangjun Bao, Xiaomei Tian

**Affiliations:** 1 Graduate Prosthodontics, Department of Restorative Dentistry, Shanghai Stomatological Hospital, Shanghai, China; 2 Graduate Prosthodontics, Department of General Dentistry, Shanghai Stomatological Hospital, Shanghai, China; 3 Undergraduate, Department of Restorative Dentistry, Shanghai Stomatological Hospital, Shanghai, China; 4 Doctor of Medicine, Department of Restorative Dentistry, Shanghai Stomatological Hospital, Shanghai, China; 5 Graduate Prosthodontics, Department of Restorative Dentistry, Xuhui District Stomatology disease centre, Shanghai, China

## Abstract

**Objectives:**

The objectives were to evaluate the degree of dye penetration of three different dental materials as esthetic veneer restorations on anterior teeth using stereomicroscope, and to provide reference for clinical choice.

**Materials and Methods:**

Sixty freshly extracted human maxillary central incisors were selected and randomly divided into three main groups of 20 each. Respectively, the teeth were prepared for veneer restoration by fiber-reinforced composite (FRC) (Everstick NET, Stick Tech), porcelain laminate veneer (PLV) (IPS Empress II(E-Max Press)) and 3M composites (3M ESPE) bonding with G-Cem resin cement (GC). After exposed to thermocycling (500cycles per day (5–55 °C, intervals: 30 s)) and immersed in 2% basic fuchsin dye solution for 24 h, the specimens were then sectioned buccolingual into three halves in a vertical plane parallel and measured dye penetration using stereomicroscope (Zeiss). The data collected was recorded by the dye penetration index (0–5) and statistically analysed using one-way analysis of variance (ANOVA) and *T*-tests using SPSS 13.

**Results:**

It is evident that the FRC group showed the lowest mean score of 0.333 mm, and the PLV group showed a highest mean score of 0.749 mm, as compared with the FRC group and the 3M composites group. Overall, when the three groups were compared for microleakage using ANOVA, there was significant difference among the groups.

**Conclusions:**

To evaluate the dye penetration of microleakage, the FRC showed a better marginal adaptability for veneer restoration. Therefore, within the limitation of this study, the use of FRC as veneer on anterior teeth can be suggested.

## Introduction

Esthetic appearance has become crucial in modern dentistry. Discoloured anterior teeth are often considered as an esthetic detraction in this modern world. As a result, veneer restoration for anterior teeth has gained so much attention, due to the growing demand for beautiful smiles and white teeth with less invasive tooth preparation. Porcelain laminate veneer (PLV) has been used by dentists for several years. It has many advantages as a restorative method, because it is aesthetically pleasing, durable and easy to use.^1,2^ However, it also has a number of drawbacks, such as tooth sensitivity, secondary caries and restoration fracture. A 10-year prospective clinical observation study reported a survival rate of 92% at 5 years, which dropped to 64% at 10 years. The main reasons for failure were large marginal defects and fractures.^3–6^ Similarly, composite veneer also has its own limitations, such as marginal staining, wear, discolouration and lower fracture resistance.^7^

Owing to the increasing importance of esthetic appearance, studies in restorative dentistry are directed towards meeting the expectations of patients and dentists. To overcome drawbacks of above materials, a high strength restorative material called fiber-reinforced composite (FRC) (Everstick NET, Stick Tech) which has been reinforced with IPN (Interpenetrating Polymer Network) composition has been recently introduced in dentistry.^8^ The previous studies evaluated the application of the FRC in the fields of post core, periodontal splint, orthodontic retainer, full crown, adhesive fixed bridge, fixed bridge, inlay fixed partial dentures, dental implant restoration and the base in removable partial denture.^9^ Even then, there still lacks a clinical application in veneer restoration.

Generally speaking, the success of any material is assessed by its longevity, sealing ability and biocompatibility in an oral environment. Microleakage is considered to be a major factor influencing the longevity of dental restorations.^10^ The purpose of this *in vitro* study is to investigate the degree of dye penetration as an estimation of microleakage of esthetic veneer restorations on the tetracycline pigmentation anterior teeth with FRC, PLV and 3M composites being introduced in clinical practice using stereomicroscope. The null hypothesis was that there were no differences among the veneer restorations with three different materials in the depth of microleakage.

## Materials and methods

### Teeth preparation

An *in vitro* experimental study was designed to assess the marginal microleakage of three different dental materials in veneer restoration. This study was performed in the Shanghai Stomatological Hospital, Shanghai, China. The research protocol was approved by the local ethics committee.

**Figure 1:**
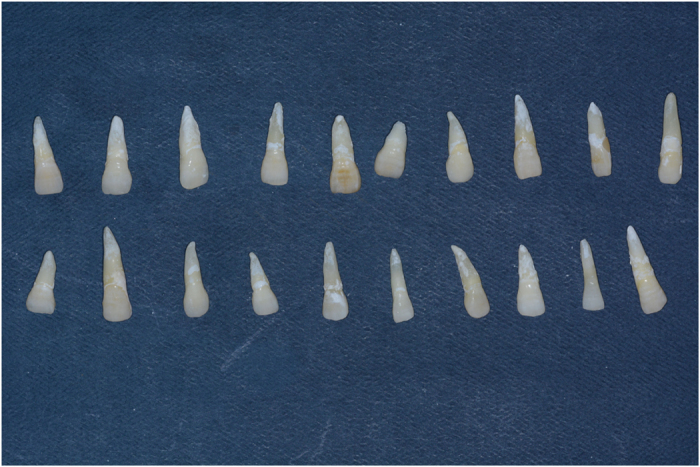
Division and restoration of samples.

**Figure 2:**
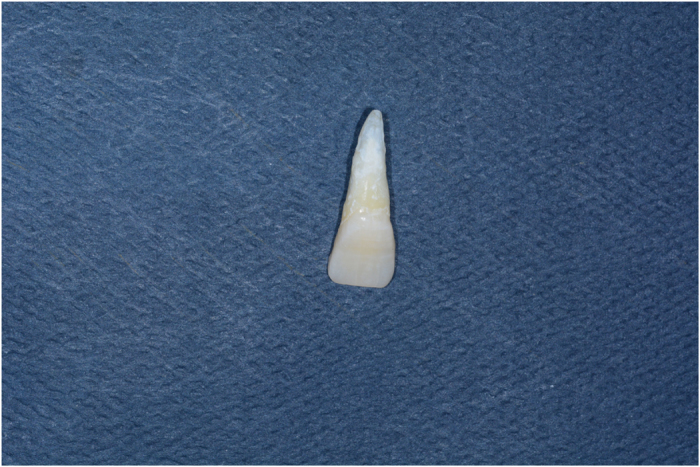
The initial state of the tooth before restoration.

Sixty human maxillary central incisors that were recently extracted within the last 3 months due to periodontal problems were selected for the study. All the teeth had no craze lines, decay, abrasion, previous restorations, structural deformities, or cervical lesions. Removal of calculus and debris was carried out by hand through scaling and then stored in normal saline, which was known to have no effect on dentinal permeability and the bond strength of cement for this study.^11^ The teeth were randomly divided into three main groups of 20 each, according to different restoration materials as follows (Figures 1 and 2):

Group 1: Microleakage seen under veneer restorations with porcelain laminate veneer (PLV) (IPS Empress II (E-Max Press)).

Group 2: Microleakage seen under veneer restorations with FRC (EverstickNET, Stick Tech, Turku, Finland).

Group 3: Microleakage seen under veneer restorations with 3M composites (3 M ESPE).

### Restorative procedure

All teeth were prepared for a veneer in the same manner by one investigator. The occlusal and buccal surfaces were reduced to 0.5 mm, respectively, with a 135° sloping shoulder finish line in the enamel. The required time for each tooth preparation was 10 min, and burs were replaced after every third tooth preparation.^12^

The impressions were obtained from each tooth using an additional polyvinyl sil oxane impression material (ESPE) and a custom-made tray (Major Tray, lot 06016A, Major Prodotti Dentori, S.p.A., Moncalieri, Italy) poured with type IV dental stone (Fujirock, GC). The veneer restorations were fabricated with three materials according to the grouping above.

**Figure 3:**
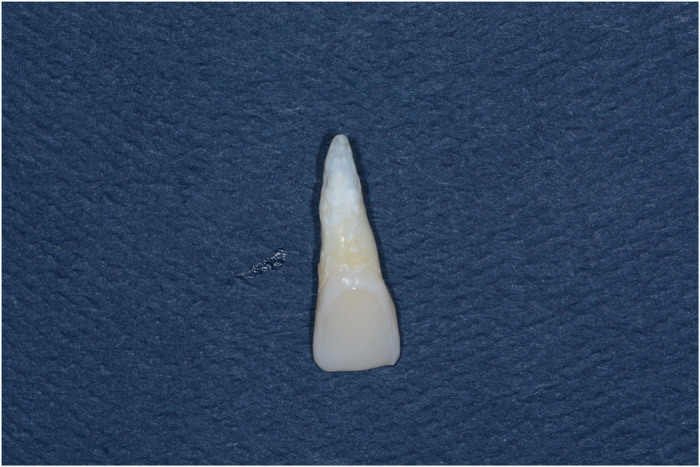
The state of the tooth after veneer restoration.

The G-Cem resin cement (GC) was applied to the intaglio surface of the veneers, and the veneers were positioned on the teeth and held in place with finger pressure. The excess cement was removed, and the laminates were exposed to light on all margins for 60 s (Coltolux 50, Coltene/Whaledent), whereas all of the margins were covered with an isolation gel (Oxyguard, Kuraray) to prevent the formation of an oxygen-inhibited layer according to the manufacturers’ instructions. All preparations were performed by the same operator at room temperature and subsequently stored in an artificial oral environment (36 °C and 100% humidity) for 1 week (Figure 3).^13^

### Evaluation of microleakage

**Figure 4:**
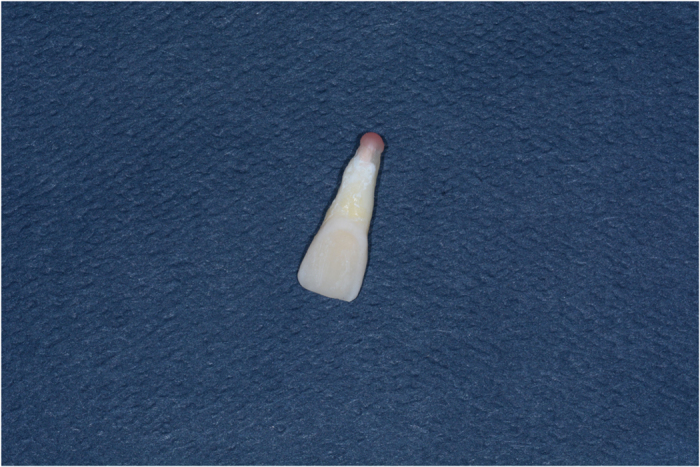
Apical sealing with the wax.

**Figure 5:**
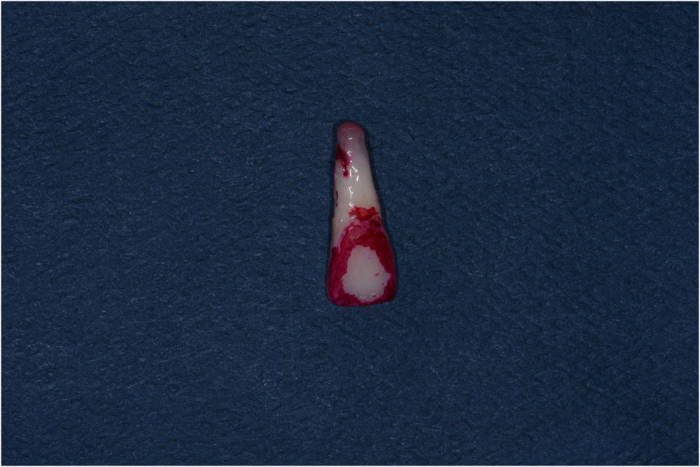
The state of the tooth after dyeing.

The specimens were then subjected to thermocycling for 500 cycles between 5±2 °C and 55±2 °C with a dwell time of 30 s in each bath and 20 s interval between baths at ambient air. Before the evaluation of the microleakage, the root apices and furcation areas were completely sealed with utility wax (Figure 4). To prevent dye penetration through the apex or dentinal tubules, all teeth received two coats of nail polish on the entire tooth surface except for the restoration and a 1 mm rim of tooth structure around the restoration margins.^14^ After sealing, the specimens were subsequently immersed in 2% basic fuchsin dye solution for 24 h, then removed from the dye, cleaned under tap water, and then left to dry for another 24 h according to manufacturer's instructions (Figure 5).

**Figure 6:**
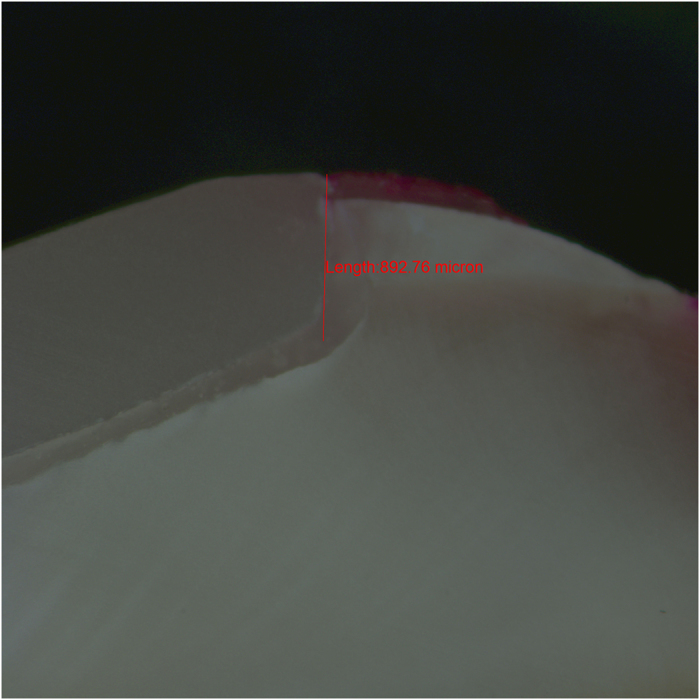
Longisection view of a tooth with porcelain laminate veneer restoration showing dye penetration.

**Figure 7:**
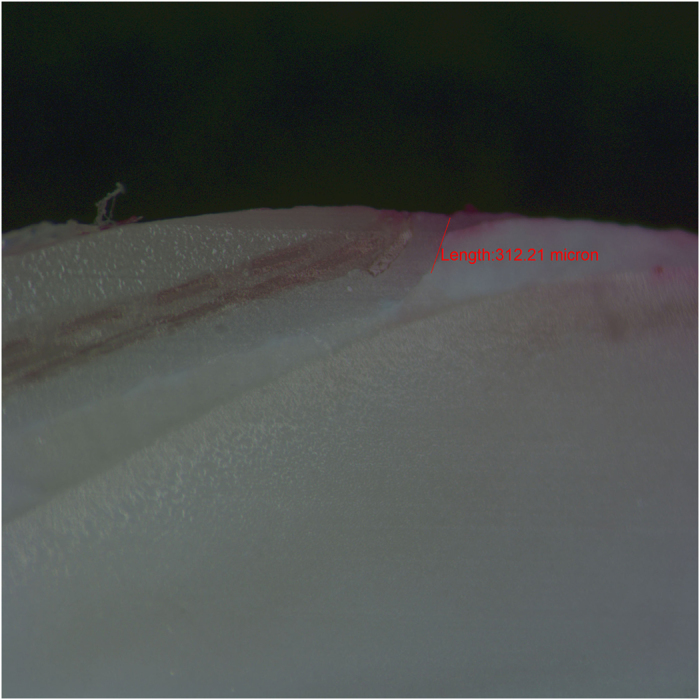
Longisection view of a tooth with everStick fibre reinforced composites resin veneer restoration showing dye penetration.

**Figure 8:**
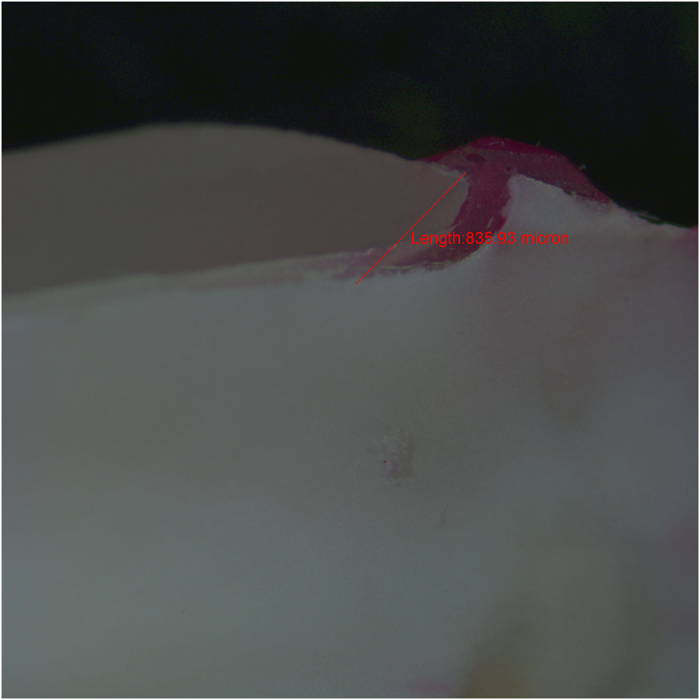
Longisection view of a tooth with 3M composites resin veneer restoration showing dye penetration.

Later on, the specimens were fixed in translucent orthodontic resins and sectioned buccolingual into three halves in a vertical plane parallel to long axis of the tooth using diamond disc at slow speed with a water-cooled diamond saw (Leitz 1600) in the buccal area.^15^ All the samples were prepared by a single trained clinician and were assessed in a blind manner by two independent evaluators under a stereomicroscope (Zeiss) at ×40 magnification based on five-point scale to evaluate dye penetration(Figures 6,7,8).^16^ The microleakage scoring was done using the method, as per Radhika *et al.*^17^

- 0=No dye penetration;
- 1=Dye penetration limited to outer half of the axial wall;
- 2=Dye penetration limited to inner half of the axial wall;
- 3=Dye penetration reach the pulpal wall;
- 4=Dye penetration beyond the pulpal wall.

### Statistical analysis

Image analysis software was used to measure the linear dye penetration in microns. All data sets were subjected to normality testing using the Kolmogorov–Smirnov test. The mean microleakage values of the groups were statistically analysed by repeated-measures analysis of variance (ANOVA) using a statistics software (SPSS ver. 13, SPSS IBM SPSS; Chicago, IL, USA). *T* test was used to compare the different groups with each other and find out which group differs significantly. The differences with *P*<0.05 were considered statistically significant.

## Results

Microleakage for each group was evaluated by stereomicroscope ×40 magnification and recorded using a parametric scale that gives a qualitative measurement of sealing effectiveness of restorative material (Table 1). As summarised in (Table 2/Figures 6,7,8), the microleakage data indicated that all the groups showed microleakage. The mean±s.d. leakage of samples from group 1 to group 3 was as follows gives a quantitative measurement (Table 2). The data collected was tabulated accordingly and was statistically analysed using one-way ANOVA (Table 2). It is evident that the PLV group showed a highest mean score of 0.749 mm, as compared with the FRC group and the 3M composites group, and the FRC group showed the lowest mean score of 0.333 mm. The *P* value was less than 0.05 indicating that the difference among the groups was statistically significant (Table 2).

## Discussion

Studies have concentrated on improving tooth-coloured restorative materials and techniques to replace the lost dental tissue and improve the aesthetic state of the anterior teeth. Ceramic veneer has been used as cosmetic restoration of anterior teeth since the early 1960s. Although it had many advantages for its aesthetic, durable and easy to use, it also had a number of drawbacks such as brittleness and easily pulled off.^18^ Therefore, demand for esthetic materials has increased markedly in recent years. High strength, greater retention and colour suitability are among the most important properties of veneer materials. Recently, a new FRC called EverStick reinforced composites, which possess specific characteristics has been developed. It’s characteristics of simple, minimally invasive application that has a higher strength, with strong cohesive force and reversibility.^19^ Because of it’s minimally invasive nature, FRC can retain the healthy tissue of tooth. Also, provided is the possibility for other therapeutic method because of the reversibility.^20,21^ In addition, it shows less polymerisation shrinkage and can be bulk cured. Elasticity and low polymerisation shrinkage stress can reduce microleakage, postoperative sensitivity, and secondary caries.^22^

The marginal adaptability of restorations is necessary for successful restoration of teeth and is therefore regarded as an important determinant of their long-term success rate. The interface between restoration and dental substrate is an area of clinical concern that can result in secondary decay and marginal discolouration.^23^ Perfect adaptation is hard to accomplish because of inconsistent physical properties between tooth structure and restorative materials.^24,25^ Factors affecting the integrity of the interface are polymerisation shrinkage, hydroscopic expansion, light polymerisation concepts, thermal cycling, occlusal stresses, bonding agent and its placement.^26,27^ In our study, the teeth preparations were of the same dimensions, and the light-curing mode was the same for all the restoration specimens. Among these factors, polymerisation shrinkage would cause hidden leakage, also known as ‘microleakage’. Microleakage is the clinically undetectable passage of bacteria, fluids, molecules or ions and was commonly observed with various restorative materials. If not treated in time, it could cause pulpal pathology.^28,29^ Microleakage had been cited as the most important reason for restoration replacement, especially in anterior veneer restorations. So controlling and eliminating microleakage has always been an important goal of operative dentistry.^30,31^ Although most previous studies had demonstrated low levels of microleakage with different materials while also reporting that microleakage did not necessarily correlate with the materials, the long-term success rate of restorations was affected by several other factors such as the size of the marginal gap and the manipulation of manipulator—the results of the present study indicated a correlation between the materials used and microleakage.^32^ In order to eliminate the effect of marginal adaptability as a confounding factor and to evaluate the sealing ability of different materials in potential open areas of restoration margins, we investigated the sealing ability of the restorations with three different materials but the same preparation and resin adhesive.

Various methods to detect microleakage were suggested, including the dye leakage method, the use of colour producing microorganisms, radioactive isotopes, the air pressure method, neutron activation analysis, electrochemical studies, scanning electron microscopy, thermal and mechanical cycling, and chemical tracers. All of the three materials utilised in this study adopted the dye leakage method and exhibited some degree of microleakage. The FRC displayed very low microleakage and more satisfactory results for sealing of marginal adaptation, which was consistent with previous reports.^33^ In addition, polymerisation shrinkage of 3M composites may have caused higher stress at the tooth-restoration bond, although further studies were needed to compare the shrinkage of 3M composites with other resin composites. These results were in agreement with the present study.^34^ Moreover, the restoration margins in the present study were placed in enamel, which had been shown to display lower microleakage compared with enamel dentin. This result appeared to support the results obtained by Bayrak *et al.*^35^ However, problems associated with the microleakage remained a concern to clinicians and may compromise the longevity of restorations.

Results of this study showed that there was statistical significant difference among the three groups in microleakage. When group 2 was compared with group 1 and group 3, it was found that FRC group exhibited less microleakage than the PLV and 3M composites group. Another important factor—which can also affect the marginal microleakage—was the distance between the light-curing tip and the resin surface. When the distance between the light-curing tip and the restoration surface was >2 mm, the light intensity was significantly reduced. This might prevent adequate polymerisation of resin composite materials and was in accordance to previous studies.^36^ As dentists, we should incessantly search for techniques that eliminate, or at least reduce, microleakage to a minimum.

The main limitation of this study was that it was performed in laboratory conditions; however, the best way to test restorative materials would be in the oral cavity. More clinical experience was needed to confirm the results in real clinical situations. Furthermore, only three types of dental materials were evaluated. However, microleakage has not been assessed in non-adapted margins compared with adapted margins in previous studies.^12^ Within the limitations of this study, it was concluded that FRC exhibited better sealing ability in compared with PLV and 3M composites. Further studies of long-term strength and marginal sealing are required to confirm the results of the present study. Furthermore, clinical trials should be conducted on the long-term efficacy of different materials for anterior teeth veneer restorations.

## Conclusions

Within the limitation of this study and regarding the results, it is concluded that all the restorative systems tested in this study exhibited microleakage. This was inevitable and irrespective to type of material being used, and the microleakage was lower in the FRC group compared with the group of PLV and 3M composites.

## Figures and Tables

**Table 1:** The qualitative measurement of sealing effectiveness of restorative materials

*Groups*	*Score*				
	*0*	*1*	*2*	*3*	*4*
Group 1(PLV)	2	3	5	4	6
Group 2(FRC)	9	4	2	3	2
Group 3 (3Mcomposites)	4	5	3	2	6

**Table 2:** Mean and s.d. values and the statistical analysis of dye microleakage of the different materials in restoration margins (mm)

*Different materials*	N	*Mean±s.d.*	*ANOVA*	
			*F*	*P-value*
PLV^a^^,^^b^	20	0.749±0.268	19.70	0.0023
FRC^a^^,^^c^	20	0.333±0.099	2.38	0.17
3M composites^b^^,^^c^	20	0.536±0.186	2.36	0.18

Abbreviation: ANOVA, analysis of variance.

a Indicate significant difference between PLV and FRC (*P*<0.05).

b Indicate significant difference between PLV and 3M composites (*P*<0.05).

c Indicate significant difference between FRC and 3M composites (*P*<0.05).
